# Psychological Distress Among Chinese College Students During the COVID-19 Pandemic: Does Attitude Toward Online Courses Matter?

**DOI:** 10.3389/fpsyg.2021.665525

**Published:** 2021-05-28

**Authors:** Yueyun Zhang, Baozhong Liu

**Affiliations:** ^1^School of Philosophy and Social Development, Shandong University, Jinan, China; ^2^Institute of Sociology, Chinese Academy of Social Sciences, Beijing, China

**Keywords:** COVID-19, online courses, psychological distress, college students, China

## Abstract

Due to the pandemic of coronavirus disease 2019 (COVID-19), taking online courses has become a “new normality” for college students. This study paid particular attention to the role of college students’ attitude toward online courses (ATOC) in shaping their psychological distress during the COVID-19 epidemic in China. Participants were from a national panel survey that has been administered before and during the COVID-19 epidemic. Besides bivariate analysis, a multivariate regression model while adjusting for a lagged dependent variable (i.e., pre-COVID distress) was estimated to show the association between ATOC and during-COVID distress. We found that respondents from a disadvantaged family background (i.e., below-college parental education, below-average family economic condition, and rural residence) were more likely to have an “unsupportive” ATOC. Moreover, both bivariate and multivariate analyses confirmed that respondents with a “neutral” or “unsupportive” ATOC had greater during-COVID psychological distress, compared to their counterparts with a “supportive” ATOC. Given the persistent spread of the COVID-19 worldwide and the profound onsite-online transition in course delivery in higher education, students’ perceptions and evaluations of the massive online courses should be carefully considered and integrated into curriculum reforms in both present and post COVID-19 situations.

## Introduction

The coronavirus disease 2019 (COVID-19), which was first reported in Wuhan, China in December 2019 and then declared a pandemic by the World Health Organization in March 2020 (World Health Organization, 2020b), is still spreading around the world with strong momentum. As of 23 March 2021, the WHO had reported over 122 million confirmed cases, including 2.7 million deaths globally since the start of the pandemic (World Health Organization, 2020a). Additionally, the COVID-19 pandemic has a psychological impact at all ages, with students and young adults being most at risk (Glowacz and Schmits, 2020; Wang et al., 2020a, b). In particular, a burgeoning body of literature has paid attention to the psychological wellbeing of college students during the COVID-19 pandemic, with evidence from China (Cao et al., 2020; Chen et al., 2020; Chi et al., 2020; Fu et al., 2021; Hou et al., 2021), the United States (Hoyt et al., 2020; Son et al., 2020; Wang and Zhao, 2020), and European countries such as Spain (Alemany-Arrebola et al., 2020), Swiss (Dost et al., 2020), and France (Wathelet et al., 2020).

Students in higher education may face unique psychological challenges associated with dramatic shifts in the mode of teaching and learning. Following extensive lockdowns and school closures, many colleges and universities around the globe have moved their courses online to maintain their academic routines. As reported in a cross-national study with a large sample of college students collected from 62 countries during May and June 2020, 86.7% of respondents claimed that their onsite classes had been canceled and substituted with online courses (Aristovnik et al., 2020). In the data we would analyze in this study, over 96% of Chinese college students reported that their schools had launched online courses and over 60% of them were taking online courses 3 h or more each day while they were interviewed in mid-March 2020. Due to the COVID-19 pandemic, taking online courses has almost become a “new normality” (Tesar, 2020) for students in higher education. This study examined the role of students’ attitude toward online courses (ATOC) in affecting their psychological distress during the COVID-19 period.

It is possible that online courses can be desirable for some students, even in the context of the COVID-19 pandemic. There has been evidence that during the COVID-19 period, some college students were worried about delays in academic activities (Cao et al., 2020), and online teaching and learning may thus be welcomed as the only way to guarantee the progression of their academic routines. In fact, for many higher education institutions nowadays, online courses are not a completely new mode of teaching and learning. The MOOCs (massive open online courses), for example, have been designed to offer a limited number of high-quality courses to an exceptionally unlimited number of students and are gaining popularity in recent years (Hew and Cheung, 2014).

However, the abrupt and massive transition from onsite courses to online courses may be considered by some students as unsatisfying and challenging, possibly due to certain beliefs that online teaching and learning cannot be as effective as onsite teaching and learning. Specific reasons could be, as revealed in some recent surveys, a lack of communication between students and instructors, a poor learning atmosphere, and an unstable internet connection (Aristovnik et al., 2020; Dost et al., 2020; Xiong et al., 2020). Furthermore, since online courses were required to be taken at home due to extensive school closures, the well-developed Internet and Communication Technology (ICT) within the campuses became out of reach. In this regard, students even from the same school can still be exposed to differential levels of internet connection and digital support which, to a large extent, was now influenced by family background (Alipio, 2020; Azlan et al., 2020). Indeed, taking online courses during the COVID-19 pandemic can be particularly challenging for students from low-income families and less-developed regions (Alipio, 2020; Azlan et al., 2020; Owusu-Fordjour et al., 2020).

Despite the growing concern about the psychological problems of college students against the backdrop of the COVID-19 pandemic (Cao et al., 2020; Chen et al., 2020; Fu et al., 2021), and despite the dramatic shift in course delivery being often assumed to be an important risk factor for college students’ mental health problems (Cao et al., 2020), we were not aware of any studies directly investigating the role of students’ ATOC in shaping their mental health during the COVID-19 pandemic.

A few recent studies offered valuable insights into this issue. A web survey of 381 university students in Jordan conducted in May 2020 found that over half of the respondents reported online distance learning as their major distressing concern, while the concern for social isolation and being affected by COVID-19 was much less prevalent (Ala’a et al., 2020). In contrast, another small-scale survey of 94 university students in China during the epidemic found that the majority of respondents regarded online courses as necessary before they were allowed to return to the campus (Pan, 2020). These inconsistent findings indicated that students’ psychological preparedness for online courses may greatly vary in different social contexts or educational settings. Furthermore, despite their insights, these studies only yielded preliminary results because of the small samples they used. As far as policy implications were concerned, these findings were not sufficient in terms of reliability and generalization to inform effective and targeted guidelines.

### The Chinese Context

China could be an important case for a better understanding of the psychological consequences of students’ perceptions or evaluations of online courses in other countries, given the persistent spread of the COVID-19 worldwide and the profound onsite-online transition in course delivery in higher education. Since a large-scale outbreak of the COVID-19 epidemic was first confronted in China, colleges in China were also first affected as compared to their counterparts in other parts of the world. On 20 January 2020, Chinese experts officially declared that the newly detected coronavirus can be transmitted from person to person. One week later, as a quick and decisive response to the outbreak of COVID-19, the Chinese government issued an order to postpone the opening of all schools and universities (Ministry of Education of China, 2020b). Another week later, the Chinese government paid particular concern to the teaching and learning in higher institutions and correspondingly initiated the so-called program “ting ke bu ting xue” which can be, in a seemingly paradoxical manner, rephrased as “suspending classes without suspending teaching and learning” (Ministry of Education of China, 2020c; Wang and Zhao, 2020). It is indeed under this guideline that the nationwide universities devoted great efforts to preparing a whole system of online courses for the upcoming spring semester. On 28 February, the Chinese government urged all schools and universities to realize that a large-scale online education in schools and universities would be a test of its whole educational system in responding to major public health emergencies, and would also be of great significance to use the information and communication technology (ICT) to promote educational reform (Ministry of Education of China, 2020a).

It is worthwhile to note that during the period from late January to late February when the number of confirmed COVID-19 cases was rapidly increasing, most college students in China were indeed still spending their winter vacation at home. When it came to March, the spring semester was gradually started for Chinese colleges, but students were still not allowed to return to the campus and were required to take their registered courses via the internet. By the time we conducted the follow-up survey in mid-March 2020, 95.7% of respondents in our sample were still at home, and 96.3% reported their schools had already launched online courses.

### The Current Study

This study focused on the relationship between students’ ATOC and their psychological distress during the COVID-19 period. Building upon prior literature, we expected to observe that holding a negative ATOC would be associated with more psychological distress. We used data from a national panel survey of Chinese college students that has been administered before and during the COVID-19 epidemic. Extending prior literature that has mostly featured a cross-sectional design, the follow-up design of the current study allowed us to adjust for the pre-COVID psychological distress to better capture the during-COVID psychological distress as a function of students’ ATOC. Our findings may serve as an important benchmark for future studies in understanding the psychological impact of online courses in other regions and societies.

## Methods

### Participants and Procedures

Participants were from a nationwide, two-wave panel survey that has been conducted in two time points: before and during the COVID-19 epidemic in China. The pre-COVID survey wave was conducted in November 2019, with an independent aim of studying students’ social values and health. A stratified, multistage sampling framework was applied. First, all the provincial regions in China were divided into seven strata, including the North, the East, the South, the Central, the Northeast, the Northwest, and the Southwest. Within each stratum, two or three colleges were chosen. Additionally, colleges from the region of East China were oversampled because of high concentration of higher institutions there. The survey included colleges of different types and ranks. As a result, a total of 20 colleges were obtained to form the primary sampling units (PSUs). Second, within each selected college, eight majors were randomly sampled, with assistance from college staff. Third, within each selected major, one class was randomly sampled from each grade. Finally, all the students in the sampled classes were invited to participate in the survey. With the technical assistance in each college, respondents were asked to finish a self-administered questionnaire embedded in a smartphone application specifically developed for the study.

We obtained a total of 17,332 participants in the pre-COVID survey wave. Of these, only half of the respondents were randomly assigned to the mental health module in the questionnaire to save space, yielding a sample size of 8,436. It is now clear that the survey was completed just before the outbreak of COVID-19. One March 11, 2020, the World Health Organization (WHO) declared that the COVID-19 can be characterized as a pandemic. It was in such a context that we conducted a follow-up survey in mid-March 2020, inquiring specifically about respondents’ ATOC and mental health. Finally, 5,070 students were successfully followed up, resulting in a response rate of 60.1%. After deleting a few cases with missing values on the related variables, the final sample size was 4,888. The study protocol has been approved by the Institutional Review Board at the Chinese Academy of Social Sciences, and informed consent was obtained from all respondents before commencing the interview.

### Measures

#### Psychological Distress

Psychological distress during the COVID-19 period was the outcome variable. It was assessed using the 10-item Kessler Psychological Distress Scale (K10) (Kessler et al., 2002), which has been widely used to screen non-specific psychological distress over a 30-day recall period. The K10 asks about the frequencies experiencing a series of negative emotional states based on a 5-point Likert-type scale ranging from 1 (none of the time) to 5 (all of the time). The total scores would thus range from 10 to 50, with higher values signifying more psychological distress. The psychometric properties of the K10 in Chinese version has been examined among Chinese college students (Zhou et al., 2008). In our study, the K10 was administered in both the pre-COVID and during-COVID survey waves. In the during-COVID survey wave, we modified the instructions regarding the time frame and asked respondents how often they experienced each symptom over a 7-day recall period instead of a 30-day recall period, because up until the survey date online teaching in most Chinese colleges had not lasted long enough for 1 month. As such, this modification can help clarify the link between students’ ATOC and their psychological distress over the period of COVID-19. The internal consistency of the K10 during-COVID wave was good, with a Cronbach’s alpha of 0.930.

#### Attitude Toward Online Courses (ATOC)

Students’ ATOC was collected using one single question: “To what extent are you supportive or unsupportive of online courses?” Response options included: (1) “highly supportive,” (2) “supportive,” (3) “neutral,” (4) “unsupportive,” and (5) “highly unsupportive.” To simplify, we collapsed them into 3 broad categories: “supportive,” “neutral,” and “unsupportive.” The collapse of the “unsupportive” and “highly unsupportive” categories was also due to fact that in our original data, the two categories had low responses, about 3.5 and 1.7%, respectively. Recent literature highlighted the Zhongyong thinking (the Doctrine of the Mean) in Chinese societies in understanding adolescents’ avoidance of extremities while reporting their attitudes (Ma et al., 2019; Lan et al., 2021). In our sample, 23.9% respondents reported a “neutral” ATOC, whereas over 70% were either highly supportive or supportive of online courses. Moreover, our single-item, self-reported measure of students’ ATOC might be subject to the social desirability bias. We would get back to this issue in discussing the limitations of this study.

#### Covariates

Covariates involved four aspects: demographic factors, family background, educational background, and pre-COVID psychological distress. Demographic factors included gender (1 = female), age in years, and whether the respondent was an only child (1 = only child). Family background factors included parental education, family economic condition and current residence. Parental education was measured by taking the highest level of education reported for either the mother or the father. It consisted of two categories: “below college” and “college and above.” Family economic condition was assessed by self-report by asking “How is the current economic condition of your family?” and included three categories: “below average,” “about average,” and “above average.” Current residence was an urban-rural dichotomous variable. Educational background included respondents’ schooling level, grade, and hours spent each day on online courses. The schooling level divides respondents into vocational students, academic undergraduates, and academic postgraduates. In terms of grade, respondents were categorized into first-year, second-year, and third-year or above. Hours spent each day on online courses included three levels: 0–3, 3–6, and 6 h or more. The pre-COVID psychological distress was also collected using the K10 scale (Kessler et al., 2002), which had a Cronbach’s alpha of 0.949, indicating high internal consistency.

### Statistical Analysis

All analyses were performed using Stata for Windows, version 12.0. Descriptive statistics were reported as means and standard deviations for continuous variables and percentages for categorical variables. Cross-tabulations with chi-square tests were firstly used to examine the distribution of Students’ ATOC across subgroups with different family background as indicated by parental education, family economic condition and current residence. We then focused on the association between students’ ATOC and their psychological distress during the COVID-19 period, using both bivariate analysis and multivariate regression. The bivariate analysis compared the difference in during-COVID psychological distress across three ATOC categories, and the significance of difference was assessed using the *F*-test. In multivariate regression, we employed a linear regression model with a lagged dependent variable adjustment to examine the relationship between ATOC and psychological distress. In this case, the dependent variable was during-COVID psychological distress and the lagged dependent variable was pre-COVID psychological distress. As such, this model controlled for the preexisting difference in distress symptoms that might confound the relationship between ATOC and during-COVID distress. Regression coefficients and 95% confidence intervals were reported. Finally, since the respondents were clustered within classes, the significance tests were based on robust standard errors allowing for correlated residuals within classes (White, 1980).

## Results

Table 1 presents descriptive statistics for all analytical variables collected in two survey waves. In terms of demographic characteristics, the respondents had a mean age of 20.0 years (*SD* = 1.8). 55.6% were female and 47.3% were only children. With respect to family background, 21.6% had a parent with a college or above education. Two-thirds of respondents considered their family economic condition as “about average.” Urban and rural residents were almost equally divided. As to educational background, the sample consisted of 42.7% vocational students, 48.5% academic undergraduates, and 8.8% academic postgraduates. First-year students, second-year students, and the rest in higher grades accounted for 35.1, 30.0, and 34.9% of the sample, respectively. At the follow-up survey time point, 39.1% of respondents spent 0–3 h, another 40.4% spent 3–6 h, and the remaining 20.5% spent 6 h or more on online courses each day. Finally, while they were inquired about their ATOC, 71.1% responded “supportive,” 23.9% “neutral,” and 5.0% “unsupportive.”

**TABLE 1:** Descriptive statistics (*N* = 4,888).

	**Pre-COVID wave**	**During-COVID wave**
During-COVID psychological distress		15.8 (6.8)
ATOC		
Supportive		71.1 (3,474)
Neutral		23.9 (1,170)
Unsupportive		5.0 (244)
Hours spent on online courses (/day)		
0–3 h		39.1 (1,903)
3–6 h		40.4 (1,969)
6 h or more		20.5 (999)
Age in years	20.0 (1.8)	
Gender		
Male	44.4 (2,172)	
Female	55.6 (2,716)	
Only child		
No	52.7 (2,577)	
Yes	47.3 (2,311)	
Parental education		
Below college	78.4 (3,834)	
College and above	21.6 (1,054)	
Family economic condition		
Below average	25.4 (1,243)	
About average	64.5 (3,155)	
Above average	10.0 (490)	
Current residence		
Urban	48.6 (2,375)	
Rural	51.4 (2,513)	
Schooling level		
Vocational students	42.7 (2,089)	
Academic undergraduates	48.5 (2,372)	
Academic postgraduates	8.8 (427)	
Grade		
1st year	35.1 (1,716)	
2nd year	30.0 (1,464)	
3rd/4th year	34.9 (1,708)	
Pre-COVID psychological distress	19.0 (7.0)	

*ATOC, attitude toward online courses.*

*Data are presented as mean (SD) for continuous measures, and% (n) for categorical measures.*

Table 2 cross-tabulates ATOC with family background variables. The proportion of respondents with an “unsupportive” ATOC was significantly higher among those from a disadvantaged background, i.e., having a parent with a below-college education (5.2%), a below-average family economic condition (6.4%), and living in a rural residence (5.4%).

**TABLE 2:** Distribution of ATOC: by family backgrounds.

	**ATOC (%)**				
**Family background**	**Supportive**	**Neutral**	**Unsupportive**	**Total (*n*)**	**χ ^2^**
Parental education					24.08***
Below college	69.4	25.4	5.2	100.0 (3,834)	
College and above	77.1	18.8	4.1	100.0 (1,054)	
Family economic condition					15.11**
Below average	67.6	26.1	6.4	100.0 (1,243)	
About average	71.8	23.7	4.5	100.0 (3,155)	
Above average	75.3	20.2	4.5	100.0 (490)	
Current residence					23.28***
Urban	74.3	21.1	4.6	100.0 (2,375)	
Rural	68.0	26.6	5.4	100.0 (2,513)	

ATOC, attitude toward online courses.

**p < 0.01, ***p < 0.001.

Tables 3, 4 focus on the association of ATOC with during-COVID psychological distress. Table 3 reports bivariate analysis by comparing mean levels of psychological distress across students with “supportive,” “neutral,” and “unsupportive” ATOC. It is obvious that students holding an “unsupportive” ATOC tended to have the highest levels of psychological distress (Mean = 18.9, *SD* = 9.6), followed by those with a “neutral” ATOC (Mean = 17.2, *SD* = 7.5), and then those with a “supportive” ATOC (Mean = 15.1, *SD* = 6.2). These discrepancies were statistically significant based on the *F*-test (*F* = 71.17, *p* < 0.001). Table 3 additionally presents the differences in pre-COVID psychological distress across three ATOC groups. Similarly, we found that respondents with an “unsupportive” ATOC also tended to have higher levels of pre-COVID distress (*F* = 31.27, *p* < 0.001). Therefore, to better determine the association of ATOC with during-COVID distress, the potential confounding effect of pre-COVID distress needs to be excluded.

**TABLE 3:** Levels of psychological distress: by ATOC.

	**ATOC**			
	**Supportive**	**Neutral**	**Unsupportive**	***F*-value**
Psychological distress during COVID (10–50)	15.1 (6.2)	17.2 (7.5)	18.9 (9.6)	71.17***
Psychological distress before COVID (10–50)	18.6 (6.9)	19.7 (6.9)	20.8 (8.8)	31.27***

ATOC, attitude toward online courses.

***p < 0.001. Data are presented as mean (SD).

**TABLE 4:** Effects of ATOC on during-COVID psychological distress.

	**Coefficients**	**95% CI**
ATOC (Supportive = 0)		
Neutral	1.663***	(1.227, 2.098)
Unsupportive	2.802***	(1.736, 3.867)
Age in years	–0.055	(–0.234, 0.123)
Female	−0.541**	(–0.893, –0.190)
Only child	0.090	(–0.295, 0.476)
Psychological distress before COVID (10–50)	0.423***	(0.392, 0.453)
Parental education (college and above = 1)	–0.056	(–0.552, 0.440)
Family economic condition (below average = 0)		
About average	–0.243	(–0.661, 0.174)
Above average	–0.004	(–0.772, 0.763)
Current residence (rural = 1)	–0.162	(–0.556, 0.232)
Schooling level (vocational students = 0)		
Academic undergraduates	–0.039	(–0.459, 0.381)
Academic postgraduates	0.361	(–0.633, 1.356)
Grade (first year = 1)		
2nd year	–0.117	(–0.570, 0.337)
3rd/4th year	0.676*	(0.059, 1.293)
Hours spent on online courses per day (0–3 h = 0)		
3–6 h	−0.536*	(–0.975, –0.097)
6 h and above	−0.683*	(–1.227, –0.139)
Constant	8.944***	(5.478, 12.410)
Observations	4, 871	

ATOC, attitude toward online courses; CI, confidence interval.

*p < 0.05, **p < 0.01, ***p < 0.001. N = 4,888.

In Table 4, we regressed during-COVID distress on ATOC, while controlling for respondents’ demographic factors, family background, educational background, and their pre-COVID distress as a lagged dependent variable. Other things being equal, greater during-COVID psychological distress was associated with a “neutral” ATOC (*p* < 0.001) and an “unsupportive” ATOC (*p* < 0.001), with the “supportive” ATOC as the reference.

## Discussion

Taking online courses has become a “new normality” (Tesar, 2020) for higher education due to the COVID-19 pandemic. In the current study, we examined the role of college students’ ATOC in shaping their during-COVID psychological distress. Our data were from a national panel survey that has been conducted before and during the COVID-19 epidemic in China. The follow-up design of our data allowed us to control for the lagged dependent variable (in this case, the pre-COVID distress) to better capture the association of ATOC with during-COVID distress.

In our sample, the overall distribution of students’ ATOC was 71.1% “supportive,” 23.9% “neutral,” and 5.0% “unsupportive.” Compared with their counterparts from an advantaged family background, respondents with a below-college parental education, a below-average family economic condition, or a rural residence, were less likely to have a “supportive” ATOC, but more likely to have a “neutral” or “unsupportive” ATOC. Although online courses are not completely new in college campuses, the COVID-19 epidemic has exposed students to differential levels of internet connection and digital support at home. Therefore, students from disadvantaged background may struggle more than others with the abrupt shifts in the mode of teaching and learning. Similarly, a web survey of Filipino college students carried out in March 2020 showed that most of the respondents answered “No” to all items in an e-learning readiness scale, and the odds of scoring low on the readiness scale was even higher among respondents from low-income families (Alipio, 2020). Our findings were also in line with a few previous studies pointing to the marked differences in the availability of digital devices and the level of computer skills between students from developed and less-developed regions (Aristovnik et al., 2020; Owusu-Fordjour et al., 2020).

Concerning the association between ATOC and psychological distress during the COVID-19 period, we employed both bivariate analysis and multiple regression, and we obtained consistent findings. The level of during-COVID psychological distress was lowest among those with a “supportive” ATOC, and highest among those with an “unsupportive” ATOC. Most notably, in our multiple regression, respondents with either a “neutral” or “unsupportive” ATOC tended to have greater psychological distress during the COVID-19 epidemic, even after adjusting for demographic factors, family characteristics, educational background, and pre-COVID psychological distress as a lagged dependent variable. One reason for this finding may be that an “unsupportive” ATOC would lower the motivation or aspiration of students to take their required online courses. Indeed, a recent web-based, cross-sectional study of college students in Jordan had revealed that having a low motivation for online distance learning could act as one of the significant risk factors for higher levels of psychological distress (Ala’a et al., 2020). Another possible reason is that an “unsupportive” ATOC may be closely linked to their increased concerns about their academic performance due to the transition to massive online courses which, as indicated in one recent study (Son et al., 2020), could have contributed to the increased risks of mental health problems in college students. More research is needed to explore these underlying mechanisms.

It has been known that late adolescence and early adulthood, when most people attend university, are peak times for onset of mental health disorders, and a few recent studies further indicated that college students and early adults experienced an increase in distress during the COVID-19 pandemic (Wang et al., 2020b; Zimmermann et al., 2020). This study added to the literature by demonstrating the significant role that students’ ATOC could play in shaping their psychological distress during the COVID-19 period.

Finally, it is worthwhile to note that in our sample the proportion of students who held a clear negative or unsupportive ATOC was as low as 5%. A possible reason was that in the case of China, the onsite-to-online transition was indeed executed in the very beginning of a new semester. As mentioned in the introduction section, the outbreak of the COVID-19 epidemic in China occurred in the winter vacation, and the higher education system had a few weeks to get prepared for the upcoming full semester of online courses. However, in many other countries such as the United States, school closure and the onsite-to-online transition came at the half-way point of the semester (Roy and Covelli, 2021). In such circumstances, students may be especially shocked by the abrupt shift in the model of teaching and learning. Another factor might be the Chinese cultural tradition. Compared to their counterparts in western societies, Chinese college students may be more likely to follow the government’s orders, espouse a “better something than nothing” perspective, and thus consider online courses as the only way of combating the undesirable academic delays due to the COVID-19 (Cao et al., 2020). In particular, a small-scale survey of Chinese students during the epidemic suggested that Chinese college students could gradually get used to taking courses via the internet (Pan, 2020). In contexts beyond China, where internet coverage or digital-device availability is lower, or students were confronted with a sudden disruption of in-class courses at the half-way of a semester, it would be very likely to witness a larger share of students with negative ATOC, which could then translate into more psychological challenges.

Findings of this study could aid in the development of finely tuned intervention strategies aimed at alleviating mental health problems of college students. First, in the process of implementing large-scale online courses, colleges and universities should keep a keen eye on how college students view the mode of online teaching and learning. Particular efforts should be made to understand the reasons why some students do not support online classes and then, accordingly make appropriate adjustments and improvements in curriculum reforms during and even after the COVID-19 period. In addition, this study also illustrated that students from disadvantaged families were relatively less likely to have a supportive ATOC. In considering that a significant number of students have been taking online classes in their own homes, home-school ties during the epidemic should be particularly strengthened to ensure that students from disadvantaged family background can be well equipped with the hardware facilities and online environment required for online courses.

### Strengths and Limitations

This study has several strengths. First, our analysis was based on a large-scale, national survey of college students. As such, we could have more confidence in making valid statistical inferences that can better inform effective policies. Second, the follow-up design of our data allowed us to employ linear regression models with a lagged dependent variable adjustment to better capture the role of students’ ATOC in shaping their mental health outcomes during the COVID-19 pandemic. To our knowledge, previous research on risk factors associated with mental health problems was often limited due to the lack of pre-COVID assessment of respondents’ mental health condition, and thus it was not certain whether the observed mental health problems were really emerging before or during the COVID-19 pandemic (Hamza et al., 2020).

There were also several limitations. The first limitation was the measurement of students’ ATOC. We collected students’ ATOC with a single question asking to what extent they supported or unsupported online courses on a 5-point Likert scale. Such a single-item self-report measurement cannot consider the social desirability on ATOC, which could possibly yield a high percentage of being “supportive” and further inflate the association of ATOC with psychological distress. In reality, students’ ATOC could be multi-dimensional, such as viewing online courses as interesting/boring, effective/ineffective, or attractive/unattractive. Future studies would benefit from better-designed instruments, such as the Semantic Differential Scale for Online Courses (Chiou, 2006). Second, the measurement of students’ during-COVID psychological distress was based on a modified K10 scale, in which we asked how often respondents experienced distress symptoms over a 7-day instead of a 30-day recall period (Kessler et al., 2002). Despite the merits of such a modification as previously mentioned, we cannot follow the commonly used cutoff in the K10 scale (Andrews and Slade, 2001; Tang et al., 2018) to further categorize the distress symptoms into “low,” “mild,” and “severe.” Third, despite the panel design, this study may still suffer from the omitted variable bias. For example, individual’s health risk perceptions during the COVID-19 pandemic have recently been documented to be related to psychological outcomes (Commodari et al., 2020; Ding et al., 2020). Unfortunately, we cannot control for these factors due to data limitation. Finally, we focused particularly on college students, and thus the results cannot be generalized to students at lower educational levels. Although the mode of online learning became prevalent for students from all educational levels, the lack of social activities (e.g., playing with peers) in the online setting may carry different meanings for students of younger ages (Commodari and La Rosa, 2020). As a result, their psychological experience related to online courses may be different from that of college students. The psychological implications of taking online courses for primary and middle school students, surely merit independent further examinations.

## Conclusion

We expanded on previous work by identifying the role of ATOC in shaping mental health of college students during the period of global COVID-19 pandemic, using data from a nationwide, two-wave panel survey of college students in China. We showed that respondents holding a neutral/unsupportive ATOC had greater psychological distress, compared to their counterparts with a supportive ATOC. Concerning the continuous spread of the COVID-19 pandemic and the profound onsite-online transition in college courses globally, higher education policies should take into consideration of students’ subjective evaluation of large-scale online courses to address their mental health problems during their adaptation to the changed mode of teaching and learning.

## Data Availability Statement

Data are available at http://www.pscus.cn/menu2.jsp?langsel=CN, with the permission of the Chinese Academy of Social Sciences.

## Ethics Statement

The studies involving human participants were reviewed and approved by Chinese Academy of Social Sciences. The patients/participants provided their written informed consent to participate in this study.

## Author Contributions

YZ and BL designed the study. BL performed data collection and data cleaning. YZ performed data analysis and wrote the manuscript. Both authors reviewed the manuscript.

## Conflict of Interest

The authors declare that the research was conducted in the absence of any commercial or financial relationships that could be construed as a potential conflict of interest.
